# Cause of Death in Neonates With Neurological Insults in the Neonatal Intensive Care Unit: Insights From A MITS Pilot Study

**DOI:** 10.1093/cid/ciab857

**Published:** 2021-12-15

**Authors:** Mary Mathew, Leslie Lewis, Athira Sreenivas, Jayashree Purkayastha

**Affiliations:** 1 Department of Pathology, Centre for Foetal and Perinatal Pathology, Kasturba Medical College, Manipal, Manipal Academy of Higher Education, Manipal, Karnataka, India; 2 Department of Pediatrics, Kasturba Medical College, Manipal, Manipal Academy of Higher Education, Manipal, Karnataka, India

**Keywords:** Autopsy, Brain, Minimally invasive tissue sampling, neonates

## Abstract

**Background:**

Minimally invasive tissue sampling (MITS) of organs has been used as an alternative to complete diagnostic autopsy in countries where refusal for autopsy in newborns is common for sociocultural reasons. There is a paucity of literature regarding the diagnostic utility of MITS of the brain after death in neonates with neurological insults, especially in India.

**Methods:**

This was a prospective, preliminary single-center tertiary care hospital study in India, focused specifically on MITS of the brain after neonatal death as a diagnostic tool to identify the various neurological insults. All neonatal deaths with neurological symptoms occurring within the first 30 days of life were enrolled, irrespective of the suspected clinical diagnosis.

**Results:**

Sixteen neonates were enrolled after death for MITS of the brain, performed for diagnostic purposes, during the study period from February 2020 to March 2021. Their gestational ages ranged from 26 to 38 weeks. All neonates had either a history of seizures and/or respiratory distress or clinical evidence of sepsis and were on ventilator support. Histopathology in all 16 neonates showed evidence of anoxia, with or without reactive astrogliosis or microgliosis. In 5 neonates with cranial ultrasound evidence of brain hemorrhage, MITS of the brain showed intraventricular hemorrhage, subdural hemorrhage, or intraparenchymal white matter microhemorrhages. Premortem blood culture–proven sepsis was seen in 9 neonates. In all cases (100%), MITS had a good diagnostic yield and was useful to establish the neurological insult in the brain.

**Conclusions:**

MITS of the brain provides an accurate and adequate diagnosis and can be an alternative to complete diagnostic autopsy for establishing the cause of death due to neurological insults, especially in low-resource settings where obtaining consent for more invasive procedure is often challenging.

Complete diagnostic autopsy (CDA) remains the reference standard for evaluating the cause of death in neonates [1]. In addition to their individual diagnostic value, autopsies can provide mortality and morbidity surveillance data, monitor public health trends, contribute information regarding the quality of care, offer valuable information in counseling parents for future pregnancies, and serve as a potential tool for research [2–4]. Despite declining trends in autopsies, multiple studies have shown that there is significant discordance between premortem diagnosis and postmortem autopsy findings, and hence there is a renewed need and interest for autopsies [5].

Given the conservative and multicultural nature of India, it is not uncommon for parents to refuse consent for autopsy owing to religious, emotional, and cultural reasons [6]. In deaths due to respiratory illness, cardiac disease, and metabolic disorders, diagnosis can be provided by interventional radiology and laboratory investigations. However, the high rates of neurological brain injury, especially in preterm and low-birth-weight infants, injury that can be confirmed by postmortem brain core biopsies, has not been studied extensively in India. There is also a global paucity in the literature regarding the utility of minimally invasive tissue sampling (MITS) of the brain in neonates, with varying degrees of sampling success [7].

Nearly all perinatal brain injury results in neonatal encephalopathy and usually presents as seizures and reduced reactivity, of which the common causes are hypoxic-ischemic encephalopathy, stroke, intracranial hemorrhage, infection, kernicterus, and periventricular leukomalacia in preterm infants [8, 9]. Of these causes, hypoxic-ischemic injury was the most common cause of damage to the fetal and neonatal brain, resulting in considerable morbidity, including motor and learning disabilities, cerebral palsy, epilepsy and seizures, or even death [10]. The term “neonatal encephalopathy” is currently used to denote all types of neurological insults and some of its risk factors include hypoxic ischemic encephalopathy, perinatal infections, placental abnormalities, metabolic disorders, coagulopathies, neonatal vascular stroke, respiratory distress syndrome, and sepsis [11–13].

MITS of the brain tissue is a feasible and less traumatic technique that uses a brain biopsy needle to obtain postmortem brain tissue cores. Because the anterior and posterior fontanelles remain open at birth, brain cores can be easily obtained through a transfontanellar approach to study various parts of the brain. MITS of the brain has been used to document bacterial, parasitic, and viral infections as well as intracranial hemorrhages, meningitis, and hypoxic ischemic encephalopathy [14–16]. This is important when parents of the deceased neonate refuse CDA, and MITS can be offered as a convenient alternative. In addition to establishing and contributing to the cause of death, providing a diagnosis may provide closure to grieving parents. Moreover, studies on MITS of the brain would ultimately help in planning better interventions and neuroprotective strategies to reduce mortality and morbidity rates and improve postnatal outcomes. The current study aimed to identify and establish the causes of neonatal death and study the pathological changes due to neurological insults, using MITS of the brain.

## METHODS

### Study Setting and Design

This was a prospective single-center study conducted in a tertiary care hospital in Kasturba Medical College, Manipal, Karnataka, India. The study included all preterm and term neonates who died because of neurological insults, respiratory distress, asphyxia, or sepsis and were on ventilator support in the neonatal unit of the pediatric department. The evidence of neurological insults included clinical signs and symptoms, such as poor feeding, weak cry, reduced reactivity or hyperalertness to stimuli, seizures, hypotonia, lethargy, coma, and suspected raised intracranial pressure. Sepsis was suspected on clinical and laboratory grounds if the neonate showed evidence of hyperglycemia, reduced movements, changes in body temperature, breathing difficulties, and abnormal heart rate. Neonates were included if their parents or guardians consented to MITS of the brain and/or CDA after their death.

The study protocol was approved by the institutional ethics committee (KH no. 347/19). A written informed consent was obtained from the parents of the neonates, and appropriate grief counseling was provided.

### MITS Procedure

The MITS procedure was performed within 1–2 hours after the death of the neonate. Anthropometric measurements, gross changes on external examination, and the presence of palpable organomegaly were documented. The posterior fontanelle (occipital approach) was identified and cleaned thoroughly with chlorhexidine gluconate 2% solution. A Bard Monopty disposable core biopsy needle (16 gauge × 16 cm; 22-mm penetration depth) was inserted in the midline, 1cm below the occipital bone between the squama and the atlas bone, advancing in different directions and depths at a 30°angle toward the orbital cavity. Six cores of brain tissue were obtained and collected in formalin jars. This procedure was repeated with the anterior fontanelle. All 12 brain cores were examined for gross evidence of hemorrhage and sent for histopathological examination.

### Histopathology of Brain Biopsy Cores

The brain tissue cores were studied at the Department of Pathology by a pathologist (MM) and analyzed for various histological patterns of injury in the brain, with an aim to establish a possible cause of death. The immunohistochemistry marker CD68 was also used to demonstrate microglial cells in selective cases. Additional histochemical stains, such as periodic acid Schiff and Grocott methenamine silver for fungi and Gram stain for bacteria, were used on tissue sections when required. Blood, cerebrospinal fluid (CSF), and eye swab sample culture reports documented before death served as confirmation of sepsis. No postmortem CSF or tissue samples were taken for microbiological culture.

### Data Review to Determine Causes of Death

For each case, clinical findings and the results of various laboratory tests were reviewed by a team consisting of a pathologist (MM), a neonatologist (LL), and a microbiologist. The cause of death were assigned and coded using the WHO *International Statistical Classification of Diseases and Related Health Problems* (*ICD-10*), *ICD-PM* classification system, and added to the registries. Biopsy results were made available in the patients’ files and on request to their parents or guardians.

## RESULTS

We enrolled 16 neonates in whom postmortem MITS of the brain was performed for diagnostic purposes. Their gestational ages (GAs) ranged from 26 to 38 weeks. All the neonates had evidence of sepsis, perinatal asphyxia, or severe respiratory distress syndrome, occurring in combination or individually. Except for a single term newborn with perinatal asphyxia (case 4), all required ventilator support. Two neonates were suspected to have bilirubin encephalopathy, and 1 had hydrocephalus with suspected ventriculitis. In 1 preterm neonate, premortem eye swab sample culture showed methicillin-resistant *Staphylococcus aureus*. The brain tissue cores obtained from the anterior fontanelle included meninges, periventricular area with germinal matrix, frontal cortex, white matter, and cerebellum, and the posterior fontanelle brain cores included cerebellum, occipital cortex, periventricular area of the fourth ventricle, and choroid plexus.

### Histopathological Analyses

The brain cores obtained were adequate for histopathological evaluation in all cases (100%). Meninges were adequately sampled in 13 of 16 cases. The brain biopsies in 13 of 16 neonates showed anoxic neurons, with or without reactive astrogliosis and microgliosis. Cranial ultrasound (CUS) reports were available in 8 cases, with abnormal findings in 5. In an extremely preterm neonate (case 3; GA, 28 weeks) with premortem *Acinetobacter baumanni* sepsis and CUS evidence of grade II germinal matrix hemorrhage (GMH), MITS brain biopsy showed GMH. In this neonate, biopsy also showed had features of meningitis, but CSF analysis could not be performed owing to improper storage of the sample. In a 1-month-old neonate (case 5) with hydrocephalus, suspected ventriculitis, and premortem *Klebsiella* sepsis, CUS showed mild periventricular flaring with subdural collection, confirmed at biopsy; biopsy also showed anoxic neurons with astrogliosis and microgliosis. In a term neonate with *Escherichia coli* sepsis and grade I GMH (case 12), biopsy showed GMH. In a preterm neonate (case 13; GA, 34 weeks) with respiratory distress syndrome and grade III intraventricular hemorrhage (IVH), brain biopsy showed similar findings of IVH with intraparenchymal microhemorrhage and anoxic neurons.

**Figure 1.**
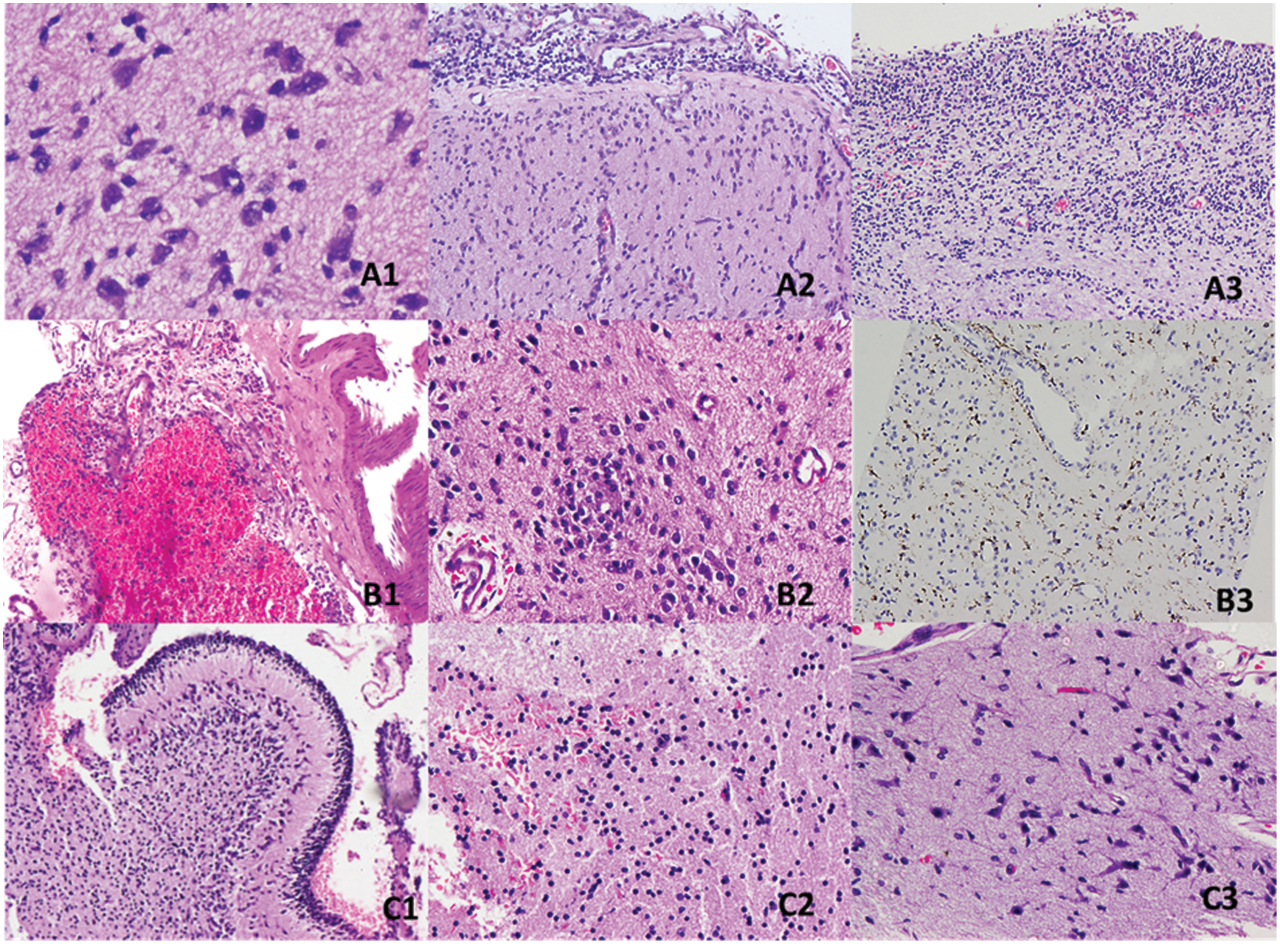
Histopathological findings in 3 selected cases. *A,* Findings in case 3, including anoxic neurons (*A1*), meningitis (*Acinetobacter baumanni* sepsis) (*A2*), and germinal matrix hemorrhage (*A3*). *B,* Findings in case 5, including subdural hemorrhage (*B1*), microglial nodules (*Klebsiella* sepsis) (*B2*), and CD68^+^ microglial cells (*B3*). *C,* Findings in case 11, including cerebellum with hemorrhage in the fourth ventricle (*C1*), white matter microhemorrhage (*C2*), and anoxic neurons with reactive astrocytes (*C3*).

In a preterm neonate (case 15; GA, 26 weeks) with *E. coli* and *Staphylococcus* sepsis and mild periventricular flaring seen at CUS, postmortem findings included microhemorrhages in the white matter, astrogliosis, and subarachnoid hemorrhage. Subdural hemorrhage was seen in an extremely preterm neonate, and subarachnoid hemorrhage in 2 preterm neonates. IVH was seen in 2 preterm neonates, GMH in 1 term and 1 preterm neonate, and intraparenchymal microhemorrhages in 5 preterm and 1 term neonate. Table 1 summarizes the clinical features, radiological findings, premortem culture reports, types of brain tissue, and histopathological findings in all study neonates, and Figure 1 depicts the histopathological findings in selected cases.

**Table 1.** Clinical Data, Investigations, and Histopathological Findings in the Study Population (n = 16)

Case	Term or Preterm (GA); Birth Weight	Symptoms and Diagnosis	CUS Findings	Infectious Pathogens Detected Before Death	Sites of Brain Tissue Obtained	Histopathological Diagnosis
1	Term (37wk); AGA	Repeated seizures from 30min after birth, septic shock, ventilator support	Normal	*Candida peliculosa* (blood)	Meninges, white matter	Anoxic neurons, astrogliosis, focal microgliosis
2	Term (37wk); AGA	Acute bilirubin encephalopathy, Rh incompatibility, seizures on d 4 of life, ventilator support	Not done	*Candida tropicalis* (blood)	White matter, occipital cortex, meninges	White matter microhemorrhage, selective neuronal and glial necrosis with loss of myelin, microgliosis
3	Extremely preterm (28wk); AGA	Multiple seizure episodes on d 6 of life, sepsis, severe RDS, ventilator support	Grade II GMH	*Acinetobacter baumannii* (blood)	Gray and white matter, cerebellum, meninges, periventricular area with germinal matrix	Astrogliosis, anoxic neurons, meningitis, GMH, microgliosis
4	Term (37 wk); AGA	Perinatal asphyxia, failure to cry at birth, HIE, multiorgan failure	Not done	Sterile	Occipital cortex, gray and white matter	Astrogliosis, anoxic neurons, and gliosis
5	Extremely preterm (28wk); LBW	Hydrocephalus with suspected ventriculitis, severe RDS, *Klebsiella* sepsis, ventilator support	Mild periventricular flaring with subdural collection	*Klebsiella pneumoniae* (blood), coagulase-negative *Staphyloccocus* (blood)	Occipital cortex, choroid plexus, meninges, periventricular area, white matter	Anoxic neurons, astrogliosis SDH, microgliosis with microglial nodules
6	Preterm (32wk, 2 d); LBW	Severe respiratory distress, pulmonary hypoplasia, ventilator support	Not done	*Escherichia coli* (blood)	Occipital cortex, meninges, choroid plexus, periventricular area, white matter	Anoxic neurons with astrogliosis
7	Preterm (37wk); LBW	Bilirubin encephalopathy, seizures, sepsis, acute kidney injury, ventilator support	Not done	MRSA (eye swab sample)	Periventricular area with germinal matrix, white matter, meninges	Anoxic neurons, astrogliosis, mild increase in microglial cells
8	Term (38 wk); LBW	Respiratory distress at birth, sepsis, severe bronchopneumonia, branding marks, ventilator support	Not done	*Candida parapsilosis* (CSF), nonfermenting gram-negative bacilli and *Staphylococcus aureus* (blood)	Occipital cortex, periventricular area, choroid plexus, white and gray matter	Astrogliosis, mild increase in glial cells
9	Term (38wk); LBW	Seizures, acute renal failure, culture-negative sepsis, ventilator support	Normal	Sterile (blood)	Occipital cortex, meninges, white matter	Anoxic neurons, microglial nodules
10	Preterm (26wk); LBW	Seizures, acute renal failure, DIC, *K. pneumoniae* sepsis, Ventilator support	Normal	*K. pneumoniae* (blood)	Occipital cortex, gray and white matter, cerebellum, meninges, periventricular area	Intraparenchymal white matter microhemorrhages with microgliosis and microglial nodules
11	Preterm (29wk); LBW	Low Apgar score, ventilator support, RDS, pneumoperitoneum, culture-negative sepsis	Not done	Sterile	Gray and white matter, periventricular area, meninges, cerebellum	Intraparenchymal white matter microhemorrhages, astrogliosis, anoxic neurons SAH, IVH (4th ventricle)
12	Term (40wk); LBW	Sepsis, aspiration pneumonia, severe RDS, ventilator support, suspected inborn error of metabolism	Grade I GMH	*E. coli* (blood)	Occipital lobe cortex, meninges, periventricular area with germinal matrix, gray and white matter	Germinal matrix microhemorrhage, anoxic neurons and astrogliosis
13	Preterm (34wk); LBW	RDS, meconium-stained amniotic fluid, DIC, pulmonary hemorrhage, IVH, culture-negative sepsis, ventilator support	Grade III IVH in 3rd and 4th ventricles	Sterile	Meninges, gray and white matter	IVH with intraparenchymal microhemorrhage, anoxic neurons
14	Preterm (34wk); LBW	Birth asphyxia, failure to cry at birth, bilateral pulmonary hypoplasia, ventilator support	Not done	Sterile	Meninges, cerebellum. occipital lobe cortical tissue, gray and white matter	Mild subarachnoid hemorrhage, anoxic neurons with astrogliosis, intraparenchymal white matter microhemorrhages
15	Preterm (26wk); LBW	Birth asphyxia, sepsis, NEC, ventilator support	Mild periventricular flaring	Coagulase-negative *Staphylococcus* (blood); *E. coli* (blood)	Meninges, cortical tissue, white matter, periventricular area with germinal matrix, cerebellum	Congested meninges, anoxic neurons microhemorrhages in white matter, astrogliosis, SAH
16	Term (39 wk); AGA	Fetal distress, failure to cry at birth, resuscitated and on ventilator support, pulmonary hypoplasia	Not done	Sterile	Occipital cortex, periventricular area, meninges, gray and white matter	Anoxic neurons with astrogliosis

Branding is traditional practice by which hot iron rods are used to burn the skin of a living person. In India, it is used as a method of treatment for diseases such as jaundice, abdominal pain, convulsions especially in children.

Abbreviations: AGA, appropriate for gestational age; CSF, cerebrospinal fluid; CUS, cranial ultrasound; DIC, disseminated intravascular coagulation; GA, gestational age; GMH, germinal matrix hemorrhage; HIE, hypoxic ischemic encephalopathy; IVH, intraventricular hemorrhage; LBW, low birth weight; MRSA, methicillin-resistant *S. aureus*; NEC, necrotizing enterocolitis; RDS, respiratory distress syndrome; SAH, subarachnoid hemorrhage; SDH, subdural hemorrhage.

## DISCUSSION

We present a prospective preliminary study of MITS of the brain undertaken specifically to document the various histopathological changes associated with neurological insults in term and preterm neonates in a referral Indian neonatal tertiary care unit. White matter injury is often due to prolonged periods of hypoxia or ischemia, infections, and inflammation, and its distribution may be focal or diffuse, resulting in astrogliosis or necrosis, depending on the severity of the insult [17]. In the current study, all cases were specifically preselected for neurological insults, and 13 neonates showed anoxic neurons, the hallmark of hypoxic ischemic encephalopathy, with or without glial necrosis or astrogliosis and microgliosis. Hypoxic changes were seen in term and preterm neonates in the neurons and glial cells in the form of anoxic neurons (red neurons) karyolysis, and karyorrhexis. Severe hypoxia and ischemia can lead to necrosis (cell death); however, in less severe insults the cell recovers or undergoes apoptosis [18]. Experimental studies show that within 5–30 minutes after the onset of hypoxia there is cytoplasmic vacuolation in the neurons and differentiating oligodendrocytes due to mitochondrial swelling. The cells affected, in order of vulnerability to hypoxic-ischemic insult, are the neurons, followed by oligodendroglia, astrocytes, and microglia in both mature and immature brains [19].

The mode of death in immature neurons is apoptosis mediated, while mature neurons demonstrate necrotic cell death [20]. In the immature preterm brain, the early-lineage oligodendrocytes, the premyelinating oligodendrocytes in the white matter, bear the brunt of injury, resulting in gliosis and periventricular leukomalacia. In term infants, on the other hand, selective neuronal necrosis may be seen in in the gray matter in 3 regional patterns: diffuse disease, cerebral–deep nuclear with prominent involvement of cerebral neocortex, hippocampus, and basal ganglia–thalamus; and deep nuclear-brain-stem disease. However, studies show that there is a blurring of this segregating concept of gray and white matter injury in term and preterm neonates and that gray matter injury can also be seen in preterm neonates [21]. This dual pattern of hypoxic changes was evident in our study in both preterm and term neonates, who showed astrogliosis as well as anoxic neurons. Microgliosis and microglial nodules are suggestive of white matter injury and can be observed in both term and preterm neonates after injurious insults such as hypoxia, ischemia, infection, and trauma [22, 23]. In our current study, microgliosis and microglial nodules were seen in both term and preterm neonates.

The entire spectrum of intracranial hemorrhage (ie, subdural hemorrhage, subarachnoid hemorrhage, intraparenchymal hemorrhage, and IVH) has been documented in term infants [24]. Subarachnoid and subdural hemorrhages are usually associated with birth trauma in term infants and are seen before labor or as late as the second week of life in preterm infants owing to hemodynamic instability. Intracerebral and cerebellar hemorrhage occurs as a result of birth asphyxia, instrumental delivery, infection, primary clotting abnormality, or congenital vascular abnormality [25]. IVH, on the other hand, is relatively uncommon in term compared with preterm infants and originates from the choroid plexus (cryptic hemangioma) or as an extension of the thalamic hemorrhage or subependymal GMH in the periventricular region, secondary to birth trauma or hemorrhage [26]. Epidural hemorrhages are rare and are associated with trauma to the skull leading to rupture of the middle meningeal artery [27, 28]. In our study, the entire range of intracranial hemorrhage, except epidural hemorrhage, was seen in both term and preterm infants. Intraparenchymal microhemorrhage was an additional observation documented in this study.

Neonatal sepsis is the second most common cause of death in India, with an incidence of clinical sepsis of 17 of 100 live births and a case fatality rate ranging from 25% to 60% [29]. In South Asia there is a predominance of gram-negative pathogens, accounting for >60% of the infections, and a low prevalence of group B *Streptococcus* compared with the West. In hospital settings, gram-negative organisms account for 63% of the pathogens, with *Klebsiella* spp. (23%) being the most common and *S. aureus* accounting for 20% of gram-positive organisms [30]. In our study, 3 neonates with *Candida* sepsis and 6 with gram negative septicemia showed microscopic features of hypoxia-ischemia, which is not specific for sepsis and can be attributed to other contributing risk factors. Meningitis was observed in only a single case.

CUS has been shown to be useful in screening, diagnosis and follow-up in high-risk neonates to predict neurodevelopmental outcomes [31]. In the current study, the CUS findings in 5 neonates who had evidence of intracranial hemorrhage were confirmed with MITS of the brain.

In an Indian study by Garg et al [32] comparing needle biopsies and CDA in neonates, adequate brain tissue was obtained in 17 cases; 1 biopsy showed meningitis, and the other 16 had normal findings. In a Turkish study of postmortem needle biopsies in neonates, successful brain biopsy specimens were obtained in 67% of cases; however, no specific pathological changes were documented in 93% of the specimens [33]. In a study by Nigam et al (India) [34], the diagnostic accuracy of postmortem needle biopsies of the brain was 50% in infants. A 2020 study by Hailu et al [7] (Ethiopia) in preterm deaths showed a brain sampling success rate of 58%. The authors expressed difficulty in diagnostic interpretation despite obtaining a fair amount of tissue, because the tissues being small, friable, and random in nature. Localized ischemic changes, kernicterus and IVH could not be detected in most cases [7].

Many studies in the past have questioned the utility and diagnostic contribution of MITS of the brain in neonates. In our study, however, the biopsy cores obtained were sufficient to establish the cause of the neurological injury, and hence we emphasize the important role of MITS and advocate for wider use of this feasible and acceptable technique, especially in low-resource settings. The current coronavirus disease 2019 pandemic has also brought MITS to the forefront, and when it is conducted with appropriate biosafety measures its findings are almost identical to those of CDA, with sufficient tissue for additional tests [35].

The current study had certain strengths. Even though MITS is a blind procedure, even a small fragment of brain core can provide useful information ,with proper technique and correlation with GA, clinical features, CUS findings, and the expected neurological insult and site of injury. Gross observation of the brain cores can also provide additional information; for example, a hemorrhagic core signifies intraparenchymal hemorrhage and hemorrhagic CSF suggests IVH or subarachnoid hemorrhage. In our study, good technical expertise and frequent discussions and collaboration with the neonatologist contributed to a high diagnostic yield.

Our study also had some drawbacks and limitations. Because this a blind procedure, the MITS technique in the brain needs to be standardized according to GA, to obtain specific tissues such as meninges and periventricular area that provide useful information and contribute to the diagnosis. The second drawback is that certain conditions, such as congenital anomalies of the brain, bilirubin encephalopathy, and lesions that affect the basal ganglia, thalamus, hippocampus, and brain stem, cannot be studied unless MITS is used in conjunction with CUS to target specific brain areas. The brain core is fragile and can lead to fragmentation and loss of tissue during processing. This technique requires good expertise and correlation with the clinical findings for maximum diagnostic yield. A comparative analysis with CDA and neuroimaging would have helped in determining the specificity and sensitivity of MITS of the brain as a diagnostic tool for neurological perinatal insults in low-resource settings.

We offer the following recommendations. A good knowledge of the anatomic structures of the brain and the sites of insults in various pathological conditions can help in interpretation, diagnosis, and etiological attribution of the various injuries occurring in the brain. Diagnostic accuracy can be increased and refined by postmortem CUS-guided MITS to target the relevant and deep-seated parts of the brain as well as by increasing the number of biopsy cores. This technique can also be an important teaching tool to guide pathology residents.

In conclusion, MITS of the brain is a feasible technique in neonates with neurological insults, especially in low-resource settings where consent for CDA is challenging or CUS cannot be performed. The current study showed that proper technique can increase the diagnostic yield and contribute to a near-accurate confirmatory diagnosis of the cause of death due to neurological insults in the brain. Its diagnostic utility can be enhanced when used in conjunction with CUS to target specific areas of neurological insults in the brain. Larger studies are required to validate the utility of brain MITS and its contribution to determining causes of death.

## Notes

***Financial support.*** This work was supported by the MITS Surveillance Alliance, funded by the Bill & Melinda Gates Foundation (opportunity/contract OPP1180554).

***Supplement sponsorship.*** This supplement is sponsored by MITS Surveillance Alliance Secretariat, led by RTI International, with funding from the Bill and Melinda Gates Foundation.

***Potential conflicts of interest.*** All authors: No reported conflicts. All authors have submitted the ICMJE Form for Disclosure of Potential Conflicts of Interest. Conflicts that the editors consider relevant to the content of the manuscript have been disclosed.

## References

[1] De Sévaux JLH, Nikkels PGJ, Lequin MH, Groenendaal F. The value of autopsy in neonates in the 21st century. Neonatology 2019; 115:21–7. 30352441 10.1159/000493003 PMC6425852

[2] McHaffie HE, Fowlie PW, Hume R, Laing IA, Lloyd DJ, Lyon AJ. Consent to autopsy for neonates. Arch Dis Child Fetal Neonatal Ed 2001; 85:4–7. 10.1136/fn.85.1.F4 PMC1721284 11420313

[3] Wainwright HC. My approach to performing a perinatal or neonatal autopsy. J Clin Pathol 2006; 59:673–80. 16803946 10.1136/jcp.2005.034504 PMC1860427

[4] Hutchinson JC, Arthurs OJ, Sebire NJ. Postmortem research: innovations and future directions for the perinatal and paediatric autopsy. Arch Dis Child Educ Pract Ed 2016; 101:54–6. 26453243 10.1136/archdischild-2015-309321

[5] Dhar V, Perlman M, Vilela MI, Haque KN, Kirpalani H, Cutz E. Autopsy in a neonatal intensive care unit: utilization patterns and associations of clinicopathologic discordances. J Pediatr 1998; 132:75–9. 9470004 10.1016/s0022-3476(98)70488-3

[6] Singh A, Bhardwaj A, Chopra M, Mithra P, Rathinam R, Siddique A. Perceptions of relatives toward medico-legal investigation and forensic autopsy: a cross-sectional survey from rural Haryana. J Med Soc 2013; 27:173–6.

[7] Hailu R, Desta T, Bekuretsion Y, et al. Minimally invasive tissue sampling in preterm deaths: a validation study. Glob Pediatr Health 2020; 7:1–10. 10.1177/2333794X20953263 PMC7457683 32923527

[8] Hagberg H, David Edwards A, Groenendaal F. Perinatal brain damage: the term infant. Neurobiol Dis 2016; 92:102–12. 26409031 10.1016/j.nbd.2015.09.011 PMC4915441

[9] Gale C, Statnikov Y, Jawad S, Uthaya SN, Modi N; Brain Injuries expert working group. Neonatal brain injuries in England: population-based incidence derived from routinely recorded clinical data held in the National Neonatal Research Database. Arch Dis Child Fetal Neonatal Ed 2018; 103:F301–6. 29180541 10.1136/archdischild-2017-313707 PMC6047140

[10] Hossain AM. Hypoxic-ischemic injury in neonatal brain: involvement of a novel neuronal molecule in neuronal cell death and potential target for neuroprotection. Int J Dev Neurosci 2008; 26:93–101. 17936538 10.1016/j.ijdevneu.2007.08.013 PMC2350216

[11] Aslam S, Strickland T, Molloy EJ. Neonatal encephalopathy: need for recognition of multiple etiologies for optimal management. Front Pediatr 2019; 7:142. 31058120 10.3389/fped.2019.00142 PMC6477286

[12] Tabares CS. Sepsis and neonatal hypoxic ischemic encephalopathy. J Neurol Stroke 2016; 4:00124

[13] Lauterbach MD, Raz S, Sander CJ. Neonatal hypoxic risk in preterm birth infants: the influence of sex and severity of respiratory distress on cognitive recovery. Neuropsychology 2001; 15:411–20. 11499996

[14] Madhi SA, Pathirana J, Baillie V, et al. An observational pilot study evaluating the utility of minimally invasive tissue sampling to determine the cause of stillbirths in South African women. Clin Infect Dis 2019; 69:342–50. 10.1093/cid/ciz573 PMC6785671 31598656

[15] Paganelli CR, Goco NJ, McClure EM, et al. The evolution of minimally invasive tissue sampling in postmortem examination: a narrative review. Glob Health Action 2020; 13:1792682. 32713325 10.1080/16549716.2020.1792682 PMC7480574

[16] Chawana R, Baillie V, Izu A, et al. Potential of minimally invasive tissue sampling for attributing specific causes of childhood deaths in South Africa: a pilot, epidemiological study. Clin Infect Dis 2019; 69:361–73. 10.1093/cid/ciz550 PMC6785686 31598659

[17] Khwaja O, Volpe JJ. Pathogenesis of cerebral white matter injury of prematurity. Arch Dis Child Fetal Neonatal Ed 2008; 93:F153–61. 18296574 10.1136/adc.2006.108837 PMC2569152

[18] Allen KA, Brandon DH. Hypoxic ischemic encephalopathy: pathophysiology and experimental treatments. Newborn Infant Nurs Rev 2011; 11:125–33. 21927583 10.1053/j.nainr.2011.07.004 PMC3171747

[19] Yang SN, Lai MC. Perinatal hypoxic-ischemic encephalopathy. J Biomed Biotechnol 2011; 2011:609813. 21197402 10.1155/2011/609813 PMC3010686

[20] Yue X, Mehmet H, Penrice J, et al. Apoptosis and necrosis in the newborn piglet brain following transient cerebral hypoxia-ischaemia. Neuropathol Appl Neurobiol 1997; 23:16–25. 9061686

[21] Miller SP, Ferriero DM. From selective vulnerability to connectivity: insights from newborn brain imaging. Trends Neurosci 2009; 32:496–505. 19712981 10.1016/j.tins.2009.05.010 PMC2743801

[22] Pierre WC, Smith PLP, Londono I, Chemtob S, Mallard C, Lodygensky GA. Neonatal microglia: the cornerstone of brain fate. Brain Behav Immun 2017; 59:333–45. 27596692 10.1016/j.bbi.2016.08.018

[23] Serdar M, Kempe K, Rizazad M, et al. Early pro-inflammatory microglia activation after inflammation-sensitized hypoxic-ischemic brain injury in neonatal rats. Front Cell Neurosci 2019; 13:1–12. 31178702 10.3389/fncel.2019.00237 PMC6543767

[24] Gupta SN, Kechli AM, Kanamalla US. Intracranial hemorrhage in term newborns: management and outcomes. Pediatr Neurol 2009; 40:1–12. 19068247 10.1016/j.pediatrneurol.2008.09.019

[25] Hong HS, Lee JY. Intracranial hemorrhage in term neonates. Childs Nerv Syst 2018; 34:1135–43. 29637304 10.1007/s00381-018-3788-8 PMC5978839

[26] Afsharkhas L, Khalessi N, Karimi Panah M. Intraventricular hemorrhage in term neonates: sources, severity and outcome. Iran J Child Neurol 2015; 9:34–9. 26401151 PMC4577696

[27] Limperopoulos C, Gauvreau KK, O’Leary H, et al. Cerebral hemodynamic changes during intensive care of preterm infants. Pediatrics 2008; 122:e1006–13. 18931348 10.1542/peds.2008-0768 PMC2665182

[28] Ballabh P. Intraventricular hemorrhage in premature infants: mechanism of disease. Pediatr Res 2010; 67:1–8. 19816235 10.1203/PDR.0b013e3181c1b176 PMC2799187

[29] Murthy S, Godinho MA, Guddattu V, Lewis LES, Nair NS. Risk factors of neonatal sepsis in India: a systematic review and meta-analysis. PLoS One 2019; 14:1–26. 10.1371/journal.pone.0215683 PMC6483350 31022223

[30] Chaurasia S, Sivanandan S, Agarwal R, Ellis S, Sharland M, Sankar MJ. Neonatal sepsis in South Asia: huge burden and spiralling antimicrobial resistance. BMJ 2019; 364:k5314. 30670451 10.1136/bmj.k5314 PMC6340339

[31] Di Salvo DN. A new view of the neonatal brain: clinical utility of supplemental neurologic US imaging windows. Radiographics 2001; 21:943–55. 11452069 10.1148/radiographics.21.4.g01jl14943

[32] Garg S, Punia RP, Basu S, Mohan H, Bal A. Comparison of needle autopsy with conventional autopsy in neonates. Fetal Pediatr Pathol 2009; 28:139–50. 19365742 10.1080/15513810902772482

[33] Celıloğlu ÖS, Celıloğlu C, Kurnaz E, Özdemır R, Karadağ A. Diagnostic contribution of postmortem needle biopsies in neonates. Turk Patoloji Derg 2013; 29:122–6. 23661349 10.5146/tjpath.2013.01162

[34] Nigam N, Kumari N, Krishnani N, Ranade R. Diagnostic yield of post-mortem needle biopsies and their spectrum: experience from a tertiary care hospital. J Clin Diagnostic Res 2019; 13:EC01–4.

[35] Rakislova N, Marimon L, Ismail MR, et al. Minimally invasive autopsy practice in COVID-19 cases: biosafety and findings. Pathogens 2021; 10:1–15. 10.3390/pathogens10040412 PMC8065952 33915771

